# How survivors of intimate partner violence experience trauma-informed care: A scoping review

**DOI:** 10.1371/journal.pone.0334623

**Published:** 2026-04-22

**Authors:** Nancy Ross, Nicole Doria, Carol McNaughton, Leslie Bagg

**Affiliations:** Dalhousie University, Halifax, Canada; University of Pretoria Faculty of Humanities, SOUTH AFRICA

## Abstract

Intimate partner violence [IPV] represents a worldwide public health concern, with most survivors experiencing the profound effects of its trauma. Survivors’ experiences of trauma-informed programs and approaches, however, are largely unknown. This scoping review therefore aimed to comprehensively map the existing literature on survivors’ experiences of IPV related trauma-informed programs and approaches. Additionally, it sought to identify gaps in the literature and highlight recommendations for trauma-informed programs and approaches related to IPV. Databases were systematically searched to identify research studies; 2,841 studies were screened, and 20 studies met inclusion for full review. IPV related programs and approaches that included one or more of six trauma-informed principles [safety; trustworthiness and transparency; peer support; collaboration and mutuality; empowerment, voice, and choice; and/or cultural, historical, and gender issues] were considered. Through employing qualitative content analysis, it was found that these programs and approaches created program satisfaction as well as positive growth and change for survivors. These changes included improved communication skills, emotional and behavioural regulation, confidence and self-worth, and health and wellness. Program dissatisfactions, barriers to entry, and recommendations for future IPV trauma-informed programs were also found to be prevalent. Implications for policy, practice, and future research are discussed.

Intimate partner violence [IPV] is a significant public health issue, affecting one in three women worldwide [1,2]. Globally, 27% of ever-partnered women aged 15 to 49 have experienced IPV in their lifetime [3]. IPV includes any behaviour by a current or former intimate partner or spouse that causes physical, emotional, psychological, sexual, or financial harm [4–6]. Although anyone can experience IPV, women are disproportionately affected [7,8]. Rates are especially high in marginalized communities, where IPV is often compounded by systemic factors such as racism, economic instability, intergenerational trauma from residential schools, and other structural inequalities [9–12].

Most survivors are deeply affected by the traumatic nature of IPV [7,13]. IPV often causes “a very specific type of trauma that is repetitive, personal, and often fluctuates between acute and chronic phases” [14, p. 321]. The core features of trauma—loss of control, disempowerment, and disconnection—closely mirror the experience of IPV [13]. While responses to trauma vary, it can lead to lasting changes in memory, emotional regulation, arousal, and cognition [15, p. 34].

Trauma may also be compounded. Childhood exposure to IPV increases the likelihood of becoming a victim or perpetrator in adulthood [7]. One global review found that individuals with four or more adverse childhood experiences were eight times more likely to be involved in IPV later in life [16]. IPV also has serious long-term health consequences. It is linked to chronic physical conditions [8,10,17–20], poor mental health—including depression, PTSD, anxiety, self-harm, and sleep disorders [18]—and a higher risk of substance misuse [21].

## Trauma-informed approach

Most IPV survivors are affected by trauma [7,13,22,23], making it essential to respond with trauma-informed services. A trauma-informed approach recognizes the widespread impact of trauma and its effects across a survivor’s lifespan [24–27]. This approach shifts the focus from asking “what’s wrong with this person?” to “what happened to this person?” [28,29, p. 4].

Trauma-informed care can be applied across diverse service settings and differs from trauma-specific interventions, which directly treat trauma [25,27,28]. While definitions vary, this scoping review uses the Substance Abuse and Mental Health Services Administration’s [27] definition as its guiding framework:

A program, organization, or system that is trauma-informed realizes the widespread impact of trauma and understands potential paths for recovery; recognizes the signs and symptoms of trauma in clients, families, staff, and others involved with the system; and responds by fully integrating knowledge about trauma into policies, procedures, and practices, and seeks to actively resist re-traumatization. [p. 9]

To guide implementation of trauma-informed approaches, SAMHSA [27] outlines six key principles that include: safety; trustworthiness and transparency; peer support; collaboration and mutuality; empowerment, voice, and choice; and cultural, historical, and gender issues [p. 10].

Wilson et al. [30] conducted the first large-scale, longitudinal study on trauma-informed care, focusing on women with co-occurring disorders and a history of violence. The intervention led to small but statistically significant improvements in trauma and mental health symptoms compared to usual care [31, p. 1213]. A more recent systematic review of IPV interventions found that both empowerment-based advocacy and cognitively focused interventions were effective, with trauma-informed approaches enhancing treatment outcomes. The authors recommended integrating trauma-informed care into IPV services [14].

Our scoping review builds on this by specifically examining how survivors experience trauma-informed programs. For LGBTQ survivors, those who perceived their services as more trauma-informed reported greater empowerment, emotional regulation, and lower social withdrawal [32, p. 6670]. Overall, there is growing support for trauma-informed care as a way to better meet the diverse needs of IPV survivors [2,30,32,33]. However, measuring its impact remains a challenge [34].

## Present study

This scoping review maps existing literature on how individuals impacted by IPV experience trauma-informed programs and approaches. It aims to identify gaps and offer recommendations to improve such services. The review is part of a larger project—*Trauma and violence-informed and family-centered responses to IPV: Charting a new course for Nova Scotia*—funded by Justice Canada. This work builds on prior research showing that current pro-arrest, pro-charge, and pro-prosecution policies can harm survivors, offenders, and families, and may discourage reporting and help-seeking [35]. By centering survivors’ perspectives, this review contributes to the development of trauma-informed, evidence-based strategies to support more effective IPV responses. We were guided by the Council of Parties’ [36] call to move from fragmented, system-centered responses toward a more relational, human-centered approach—one that “reorient[s] systems around human experience” [36, p. 379]. To achieve this, it is essential to listen to survivors. As Kulkarni stresses, IPV programs must “seek survivor input in all aspects of service planning, delivery, and evaluation” [33, p. 7] and promote meaningful participation.

## Methods

The main objective of this scoping review was to map the available evidence on individuals’ experiences of IPV related trauma-informed programs and approaches [37]. Through mapping this evidence, we aimed to identify the implications for research, practice, and policy in relation to this topic [38–40]. This scoping review was guided by Arksey and O’Malley’s research framework, which proposes methodical stages of conduct including identifying the research question, identifying relevant studies, selecting relevant studies, charting the data, and summarizing the findings [38].

### Identifying research question

The research question for this scoping review is: ***How do individuals, impacted by IPV, experience trauma-informed programs and approaches?***

### Identifying relevant studies

The literature search for this scoping review was designed in June 2022 in collaboration with a medical librarian at the Maritime SPOR SUPPORT Unit. It contains controlled vocabulary terms and keywords related to IPV and trauma-informed response and care. The search strategy was peer reviewed by another medical librarian using the Peer Review of Electronic Search Strategies [PRESS] guidelines [41]. The search strategy was designed and tested in MEDLINE All [Ovid], and once finalized was translated to PsycINFO [EBSCOhost], Social Work Abstracts [EBSCOhost], Gender Studies Database [EBSCOhost], Social Services Abstracts [ProQuest], and Sociological Abstracts [ProQuest]. No limits were applied to the search and all searches were run from inception to present. Table 1 details the MEDLINE All search strategy. All database searches were executed on June 28, 2022.

**Table 1:** Ovid MEDLINE All search strategy, subsequently translated to other databases and executed on June 29, 2022.

1	((partner? or spouse? or spousal or husband? or wife or wives or boyfriend? or girlfriend? or domestic or gender based or gender or famil*) adj2 (violen* or abus*)).ti,ab,kf.
2	(IPV or battered wom?n).ti,ab,kf.
3	exp Intimate Partner Violence/
4	Domestic Violence/
5	Gender-Based Violence/
6	Battered Women/
7	or/1–6
8	((trauma or violence or adverse child* experience? or adverse child* event?) adj (informed or respons* or based or care)).ti,ab,kf.
9	7 and 8

### Selecting relevant studies

Inclusion and exclusion criterion (Table 2) were established by the research team to guide the selection of relevant studies [38]. Research studies were included if they had first person accounts of individuals who had experienced IPV and participated in related trauma-informed programs and approaches. For the purposes of this review, programs were defined as interventions, supports, or resources designed to assist survivors and prevent or address intimate partner violence. These initiatives encompassed a variety of services, including counseling, legal assistance, educational workshops, and shelters for victims, aiming to provide comprehensive support and/or intervention. Approaches to IPV intervention refer to broader strategies and/or methodologies used to address IPV and range from prevention and education efforts to therapeutic interventions and assessment tools. There is often overlap between programs and approaches as they both share the common goal of supporting survivors and addressing abusive behaviors within intimate relationships.

**Table 2:** Inclusion and exclusion criteria for selected articles.

Inclusion Criteria	Exclusion Criteria
• First person accounts of individuals impacted by IPV and their experience related to trauma informed programs and approaches • Programs and approaches that were trauma informed/included at least one of the six guiding principles to a trauma-informed approach: safety; trustworthiness and transparency; peer support; collaboration and mutuality; empowerment, voice and choice; cultural, historical, and gender issues • Qualitative findings (including qualitative components of mixed methods studies) • Published in English	• Research articles that did not include first person accounts of individuals impacted by IPV and their experience related to trauma informed programs and approaches • Programs/approaches that were not trauma informed/did not include any of the six guiding principles to a trauma-informed approach: safety; trustworthiness and transparency; peer support; collaboration and mutuality; empowerment, voice and choice; cultural, historical, and gender issues • Quantitative findings • Not published in English • Commentaries and editorials • Literature/systematic/book reviews • Conference proceedings and dissertations

Programs and approaches were considered trauma-informed if they included at least one of the six guiding principles included in a trauma-informed approach as outlined by SAMHSA [27]: safety; trustworthiness and transparency; peer support; collaboration and mutuality; empowerment, voice, and choice; and cultural, historical, and gender issues. Determination of whether an article included one of these guiding principles depended on how the program/approach was described in the article. Explicit mention of a guiding principle or the intentional portrayal of the program/approach reflected its adherence to the principle. For a detailed explanation of each principle, we relied on SAMHSA’s manual: Concept of Trauma and Guidance for a Trauma-Informed Approach [27].

Given that we were interested in first-hand experiences of IPV related trauma-informed programs and approaches, only research studies with primary qualitative data were included. Mixed-methods studies were reviewed, however, only the qualitative components were considered and included if relevant. Studies from all countries, as well as studies published at any date, were eligible for inclusion if published in English. Reviews, conference abstract proceedings, dissertations, and literature that did not include empirical data [commentaries, editorials, book reviews] were excluded.

Screening was conducted independently by two reviewers [ND, CM] based on the above inclusion and exclusion criterion. All citations from the database searches were imported into Covidence for screening, and duplicate records were automatically removed. Articles were first screened at the title/abstract stage and, if included, screened at the full-text stage. Reviewers met regularly throughout the screening process to resolve any screening conflicts [42,43]. Reviewer one [ND] is a PhD in Health student whose dissertation research focuses on sexualized violence against women. She has previously conducted several scoping reviews. Reviewer two [CM] is a Master of Social Work candidate whose thesis explored trauma and violence informed practice. She has worked as a social worker for five years in a variety of community development and residential youth care settings. If voting conflicts could not be resolved by the first two reviewers, a third reviewer [LB or NR] was consulted to determine final inclusion or exclusion [42].

### Charting the data

The research team collectively determined that the following data be extracted and charted from each included study: first author/year, title, aim of study, method, participants, location, IPV trauma-informed program and/or approach, and included trauma-informed principles. ND and CM extracted data independently using a data-charting table in Microsoft Word. ND then collated the data and LB checked the extraction table for consistency and alignment with the research question [38,42,43]. Please see Appendix A for extracted data.

### Summarizing findings

Qualitative content analysis was employed to categorize and map the findings of included studies [43,44]. Our content analysis was guided by Elo and Kyngäs’ three phases: preparation, organizing, and reporting [45]. Two reviewers [ND & CM] completed this process by familiarizing themselves with the data; organizing the data through open coding; creating categories/a coding framework that focused on individuals’ experiences of the programs and approaches; and reporting the overall experiences of individuals. Inductive content analysis was used to derive our findings from the data, as to our knowledge there are no previous studies that explore this phenomenon [45]. The two coders engaged in discussions to resolve any discrepancies in their independent coding. Content analysis was completed in Microsoft Excel.

### Findings

A total of 4,670 studies were identified from the databases searched [MEDLINE, PsycINFO, Social Work Abstracts, Gender Studies Database, Social Services Abstracts, and Sociological Abstracts]; 1,829 duplicates were removed. At the title/abstract stage, 2,841 studies were screened and 2,756 studies were excluded. There were 85 studies screened at the full-text stage and 65 studies were excluded. Studies were excluded for not including a trauma-informed program or approach [n = 37]; wrong participant sample [n = 16]; no qualitative data [n = 6]; wrong source type [n = 3]; not being about IPV [n = 2]; and/or not published in English [n = 1].

**Fig 1:**
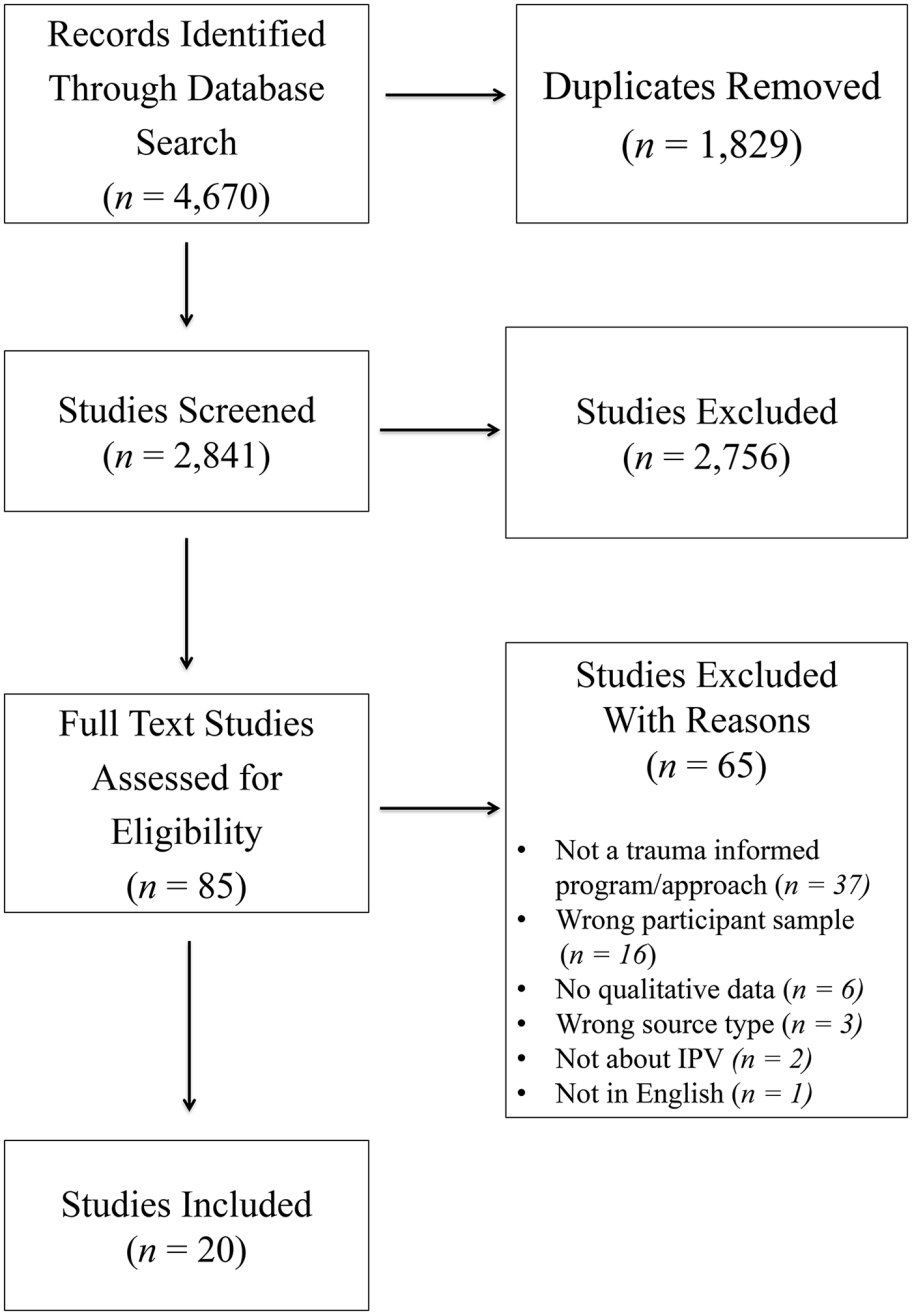
PRISMA flowchart of study selection process.

A total of 20 studies were included in the final data set [see Fig 1 for PRISMA diagram] [46]. Of these studies, ***12 were qualitative*** [47–58], ***four were mixed methods*** [59–62], and ***four were evaluations*** [63–66]. Study participants were diverse in age, racial/ethnic/socioeconomic backgrounds, sex, gender, relationship status, and location; the studies took place in eight different countries including Canada, United States, Australia, United Kingdom, South Africa, Nepal, Vietnam, and Lebanon.

### Programs and approaches

All programs and approaches incorporated trauma-informed principles, with most incorporating more than one principle. The principles included ***safety*** in 14 studies [47–52,54,56,58–64]; ***trustworthiness and transparency*** in five studies [47,48,51,56,60]; ***peer support*** in nine studies [48,51–54,57,62,63,66]; ***collaboration and mutuality*** in nine studies [48,50,51,54–56,58,64,67]; ***empowerment, voice, and choice*** in 14 studies [47,49,51–54,56–58,61,62,64,66,68]; and ***cultural, historical, and gender issues*** in 10 studies [47,48,51,53,55,57,58,63,66,67].

The majority of the programs and approaches [n = 13] explored participants’ experiences with trauma-informed IPV related ***interventions*** [48,50–54,56,59,62–64,66,68]. The remaining seven studies included the exploration of two trauma-informed ***housing programs*** [49,61], two trauma-informed ***models of service*** [58,67], a trauma-informed ***assessment tool*** [47], a trauma-informed ***training course*** [57], and a trauma-informed ***mobile app*** [55] for individuals impacted by IPV. More than half of the programs [n = 11] were delivered by clinicians and/or professionals of various backgrounds [48,50–56,64,67,68], and the remaining programs [n = 9] were delivered by program staff or volunteers who received the necessary training and/or had relevant expertise.

The programs and approaches were targeted at women, men, and/or families who had experiences of IPV. ***Women*** were the sole participants in 12 studies [47–50,52–54,56,58,59,61,68]. ***Men*** were the sole participants in one study, which focused specifically on male perpetrators of IPV [63]. There were both ***men and women*** in four studies [57,64,66,67]. One study had mostly women participants, with one participant identifying as ***bi-gender*** [51]. One study included ***mothers and their children*** [62]. One study did not specify the gender of their participants [55]. Further, three studies focused on the ***family unit*** [7,64,66], three studies focused on ***women with children*** [49,61,62]**,** two studies focused on ***youth*** who had experiences with ***sexual exploitation*** and/or ***homelessness*** [51,54], one study focused on ***postpartum women*** [50], and one study focused on women who were ***veterans*** [68].

### Conditions for program satisfaction

All studies reported some level of participant satisfaction, which was strongly linked to feelings of trust, safety, support, and empowerment—key elements of a trauma-informed approach. Participants described how these factors created environments conducive to healing and recovery from IPV. Safety was a central theme in program satisfaction. Nine studies explicitly aimed to promote safety or deliver services with a sensitivity to physical, emotional, and/or cultural safety [47,50–52,56,58,59,61,64], while others embedded safety through the setting itself [48,49,62,63] or through small group formats [ ].

Thirteen studies identified safety—whether physical, emotional, or cultural—as a condition for program satisfaction [e.g., 47,57,68]. Feeling safe helped participants engage in the programming and supported their healing process: “[Open Dialogue] was happening at a place I feel safe...if it was somewhere else where I wasn’t too sure of [safety]... I’d probably feel a bit sceptical” [48, p. 142]. Participants described the importance of experiencing both physical and emotional safety:

It is wonderful, I feel more safe.... I feel so safe. So before when I went to sleep.... I was not sleeping well because I’m thinking somebody will come attack me, especially my abuser. So now I feel so comfortable in here because you know we have cameras, we have a safe place, safe doors, you know, things like that. [49, p.535]

Another participant echoed:

It was just a very supportive environment. I felt very safe there and I felt it was ok for me to feel afraid or angry, um, and it really helped validate everything I was feeling, which also was very important because it’s very isolating. [56, p. 423]

Trustworthiness and transparency were fostered in several ways: by intentionally building trust [51,56], encouraging group dialogue over time [57], prioritizing openness [48], and using trusted staff to explain assessments and their purpose [47]. In eleven studies, participants described how trust enhanced their satisfaction with the program. This included increased trust in support systems [67], within group settings [48,51,53,54], and in their relationships with service providers [47,50,58,59,61,64]: “He [practitioner] was talking with a great deal of confidence about his field and about previous experience in cases. He did a very good job building my confidence in him and in the programme” [64, p. 547].

Peer support was most commonly cultivated through group-based interventions or support groups that encouraged and facilitated discussions amongst members [48,53,54,57,63,66]. Peer-mentorship [54], peer-lead after-care programs [63], and invitations to share resources with peers [52] were also utilized. Peer support contributed to participants’ satisfaction with programming in seven studies [48,49,51,53,54,57,62]. Overall, participants highly valued sharing with and hearing from group members who had common experiences:

I came here and I thought I was going through a really bad situation, but then you hear someone else going through something. It makes it easier...you can relate. A lot of women when they feel afraid or embarrassed and they hear someone else...they feel they can relate and it makes them more comfortable. [53, p. 60]

Participants also enjoyed experiencing a sense of belonging and having opportunities to build community and social connections. Participants shared: “there is now something that unites us together, and it feels like each of us found himself in a role specific to him [within the group]” [57, p. 58]. An individual differentiated the support she received from peers as compared to professionals, illuminating the importance of peer support:

I feel like I can just be very, very open and honest about everything and anything. So, that’s a good thing. Cause, sometimes - like I’m an open book but sometimes I don’t like to tell like, professionals, like workers and stuff like my full story or anything. But, when it’s there, they’re like your friends...They’re just like regular people who are in the group with you. [51, p. 829]

Peer support provided opportunities for participants to validate each other, engage in shared vulnerability, and experience non-judgemental acceptance: “[I]was able to just talk freely and not be judged.... Everyone was just like, hearing you out.... You have a voice” [54, p. 1304]; “It’s a non-judgmental zone” [51], p. 827].

Collaboration and mutuality were incorporated into programming through collaborative program design processes [55,64,67], tailoring group content to participants’ needs and preferences [51,54], and emphasizing partnership and problem-solving processes between service providers and users [48,50,58]. Collaboration and mutuality were described as contributing to program satisfaction in eight articles [48,50,51,53,54,58,61,64]. Participants described appreciating opportunities to engage in cocreation [54] and coproduction [51] within group processes. In one program, a collaborative approach to dialogue minimized the power differential between professionals and service users: “instead of the spotlight being on yourself it’s actually, it’s like a roof lit up... you don’t feel interrogated” [48, p. 143]. Participants also valued collaborative relationships with professionals providing support so that they could work together to meet their goals:

Well, for instance, for this new place, we’ve been calling around, me and my women’s advocate have been…She would do half of them and I would do the other half just to see if we got anywhere and we came across two places that were accepting people by next month. [58, p. 12]

Another woman echoed: “it’s quite nice to, I don’t know, to have a relationship because they’re not just your worker, you end up forming a bond, like a relationship with them, which is quite nice” [64, p. 547].

Empowerment, voice, and choice are core principles of trauma-informed care. Nine programs in this review explicitly aimed to promote empowerment [49,52–54,56–58,64,68]. Several incorporated flexible options that allowed participants to make choices—such as selecting activities, influencing topics, or setting the pace [47,51,57,61,62,68]. One program focused specifically on economic empowerment [66]. Seven studies reported that these elements enhanced program satisfaction [47,48,51,52,58,62,68]. Participants valued having input—such as choosing what to participate in, influencing the session structure, and controlling what and when they shared. As one participant explained, the benefit came from having a structured yet flexible session model:

I liked that we had a menu. Every week I got to pick because then I don’t have to sit there and wonder, ‘okay, what do I want to talk about today.’ She gave me options. If I didn’t want to deal with the feelings today and instead deal with coping with stress, that option and choice was very good for me. [68, p. 11]

Participants also expressed feeling empowered through supportive relationships that build confidence:

They help focus you on certain goals that you need met and a lot of it really is empowering. If you’ve got somebody telling you forever you can’t do anything or you don’t do anything right or–a lot of people fall into that and when they’re away from it, they don’t know where to start from and it does give them a positive reinforcement. Yes, you’re capable of doing this for yourself or getting employment to feel better about yourself, not just for money, but it helps redefine you or give you somewhere to start from. [58, p. 13]

One individual shared that being invited to share resources with others was empowering: “it makes me actually feel like I have a lot of power to help somebody... It was good to know that I had it [small educational card] on hand” [52, p. 10].

The most common ways that cultural, historical, and gender issues were attended to were through adapting or tailoring programming to be specific to participant’s cultural contexts [53,57,63,66]. A further strategy included adopting a feminist or gendered lens to programming [48,58,67]. Cultural, historical, and gender issues were mentioned as promoting participants’ program satisfaction in four articles [51,54,57,67]. Participants shared about the value of being in a female-identified group and how it allowed them to speak freely about gender issues [51,54]. One individual shared:

The open dialogue that was had within the program allowed women to embrace each other. Whether it be who we are in our sexuality, our gender, whether we’re transitioning into a new stage of life or a new gender, or we are growing and developing. We were able to have that open dialogue with just women, and it’s a crazy-ass dynamic... it was really inspiring because you saw your sisters change and grow and you’re like, “Oh, look at you!” So, it definitely helped me to see how community develops and how friendships develop. [54, p. 1306]

In one article, male participants described how it was influential that explanations about IPV were “culturally grounded in religion” [57, p. 56] as this helped participants to accept them.

### Participant growth and change

Participants experienced a variety of self-reported growth and change through their participation in trauma-informed programs. In 14 studies, individuals spoke to the knowledge they acquired from program participation and how such knowledge directly influenced positive shifts in their behaviour [49–54,56,57,61–63,66–68].

#### Increased awareness.

Increased awareness was a key behavioural change reported in 13 studies [47,49–51,53,54,56,57,59,62,63,67,68]. Participants described gaining greater self-awareness and a deeper understanding of gender-based violence [GBV] and victimization. They became more able to recognize both their own behaviours and those of others, identifying unhealthy patterns and understanding the dynamics of abuse. Psychoeducation helped participants distinguish between healthy and unhealthy gendered interactions and reject GBV as unacceptable. One participant reflected: “it just made me more aware. Like, now I know what’s not acceptable and what’s acceptable when it comes to gender-based violence. I think that’s a positive. That’s a good thing” [54, p. 1305]. Similarly, another participant shared: “when I came to counsel cases in which the wife is very talkative, I would think that maybe the husband was right to beat such a wife... after being trained, I think that all people have rights” [67, p. 1427]. Women also began to identify gender inequality as a root cause of GBV, and some recognized the possibility for change within their abusive relationships.

#### Communication skills.

Participants in six programs reported learning valuable communication skills [51,54,56,57,61–63,66,68]. These included active listening, speaking with care, using calmer language, and asserting themselves respectfully. One participant explained: “I communicate better, I can be angry but am able to express myself without hurting others in a calm respectful manner” [63 p. 1407]. Improved communication also strengthened family relationships: “communication with my parents has been a bit easier. I’ve noticed I can actually talk to my mother about issues which I was never able to do before” [54, p. 1307] and “our relationship is much better these days. We talk to each other properly” [66, p. 16]. Participants gained confidence in expressing themselves: they learned “how to control and express [themselves] by using skills like assertiveness” [63, p. 1408]. This growth encouraged women to speak up and seek help: “I learned that I am not alone. That I cannot stay quiet; I must talk about my problems to feel better” [53., p. 61]. Men also became more open, using group discussions to share emotions and strengthen dialogue with others: “these groups allowed us to open up inside and empty what’s been bottled in our hearts” [57, p. 58]. These communication skills also helped participants better express and manage overwhelming emotions.

#### Emotional and behavioural regulation.

Improvement of emotional and behavioural regulation was a behavioural change individuals experienced because of eight of the programs [49,50,53,54,56,57,62,63]. Participants stated: “I am learning to recognize my emotions” [63, p. 1407] and “I am able to control my anger and identify my emotions” [63, p. 1407]. Another individual shared:

last year... I was getting mad at people... without really thinking first. I don’t really lash out at people like that anymore... I usually, I try to approach people more calmly like even if I am mad. [54, p. 1309]

Overall, individuals developed a strong grasp of emotions and gained a better understanding of how to handle and regulate challenging emotions and behaviors. They also learned how to appropriately respond to unforeseen circumstances and became more adept at identifying and utilizing skills during stressful moments. Participants acquired effective tools and strategies to take proactive measures to minimize and manage triggers, defuse tense situations, and decrease anger, anxiety, and irritability: “taking a deep breath goes a very long way. Understanding when I become emotional that my actions or thoughts are never the best and my quality of judgment goes down” [63, p. 1408]. Another individual expressed:

The thing that sticks out the most is I learned mindfulness and how to control the anxiety attacks because I was having them so regularly and I didn’t want to be on Ativan or medication. So the mindfulness was huge for me and how to breathe and techniques like that so that I wasn’t stuck on medication. [50, p. 312]

Other individuals described how, when upset, they would now go for walks or engage in positive self-talk. For example: “I can say to myself, ‘Hey, I can’t do anything about it.’ It’s a lot easier for me to say that now than it was a year ago” [54, p. 1309].

In two studies [57,63], men experienced a decrease in anger and irritability, including towards their spouses and children. Men shared: “I was quite irritable, and I would take it out on my wife, now I understand that I shouldn’t treat her like that, not like before, where I would be really irritable with her” [57, p. 58]; “I manage my anger better, I manage better my triggers, lower my voice” [63, p. 1407]; and “I haven’t had outbursts of anger since starting the course” [63, p. 1407]. One study [62] focused on children, in which they experienced similar behavioural change. The children learned “when we are angry or sad what we can do” [Mbali, 13 yr female, Johannesburg] [p. 6]; “how to be nice and how to feel calm in our body” [Melissa, 9 yr female, Brooklyn] [p. 7]; and “when you mad it helps you to be happy; it helps you not to be sleepy and it is good to have fun” [Trinity, 7 yrs female, Brooklyn] [p. 7].

#### Building confidence and regaining self-worth.

Building confidence, independence, and a sense of self-worth was discussed in ten studies [48–50,53,54,56,57,61,66,68]. Participants reported feeling more confident and capable, which boosted their self-efficacy and led to a stronger appreciation for their self-worth and capabilities. Participants commented that they gained “a lot more self-love” [54, p. 1306] and belief in themselves: “I believe in myself more than I did before…I think I can do things again” [54, p. 1306]. Another individual shared how the program created a sense of agency and helped expand her confidence in managing problems: “it made me think that I could work through things myself” [48, p. 143]. Through reinforced confidence, women were better able to stand up for themselves:

Past relationships I’ve been in, I’ve never really had a say in them. I used to stay quiet in the relationships. But, now I’m just like, you know what? I got to do this for myself. Not just for myself, I’m having a baby. I need to do it for the baby too…They really helped me... like really standing up for myself. [51, p. 828]

This journey of self-discovery led to a greater sense of confidence, self-esteem, and increased control over the participants lives.

#### Health and wellness.

In six studies, participants described how programs supported their health and wellness [49,54,56,57,61,68]. Many reported adopting self-care and stress management strategies [49,54,57,68], while others noted improvements in mental and emotional health, including reduced symptoms and greater emotional stability [54,56,61,68]. Participants also experienced positive physical health changes and began prioritizing their well-being [54,56,68]. These outcomes contributed to greater psychosocial wellness [54,57,68], improved family wellness [61], and enhanced quality of life [56].

Participants shared: “I would say that I’m a lot better—like, healthier diet and eating habits and sleeping and general self-care has improved... not beating myself up over what I’m feeling. So that’s really nice” [54, p. 1307]. Other participants shared that “[RISE] really impacted my physical health so much. I try to make plans to workout more or exercise or do things that were more towards my physical wellness. Emotionally, RISE kind of helped me to stabilize my emotional roller coaster on some days” [68, p. 10], and

I don’t have the typical stress-related IBS anymore. My, um, pain level–I’m not in constant pain from depression and anxiety and sleeping, I mean, I am eating trying to be healthier. I can because I am allowed to because I can make my own choices. So, you know, there’s a vast improvement in just overall generally. [56, p. 423]

### Program dissatisfactions

Participants in eight studies expressed dissatisfaction with aspects of the programs [49,51,52,57–59,61,67]. In six studies, participants found some supports or resources “underwhelming” [59, p. 962] or felt that limited programming failed to meet their needs [49,52,57,59,61,67]. Additional concerns included difficulty engaging men who use violence [67], culturally irrelevant gender content [57], and discomfort when services felt compulsory rather than voluntary [58]. In one study, participants criticized wait lists, short program duration, visitor restrictions, and interpersonal conflicts with staff or peers [61]. Another study highlighted the emotional toll of group discussions, particularly when participants felt unprotected from triggering material [51]. Participants believed this issue could be better managed by facilitators and service providers:

She [service provider] has known me before that too. And, she knows what could be triggering for me. She knows what can hurt me and what sensitive topics can hit... And, it was just kind of really shocking that she was not able to prevent that from happening for me in the group. [51, p. 828]

Further, barriers to program entry were addressed in three studies [57,64,67]. Barriers included stigma; resistance and sensitives around discussing IPV; and fears related to divorce, custody of children, social disproval, and the inability to preserve appearances. Some of these barriers can be addressed: Domoney et al. discussed how they removed the language of ‘perpetrator’ and ‘victim’ during their sign-up process because of feedback received on stigmatizing language [64].

### Recommendations

In nine studies, participants made recommendations to improve the IPV trauma-informed programs and approaches they participated in [47,50,52,55,58,59,61,67,68]. Among the recommendations, commonalities were found concerning the importance of time and building support networks. It was important to participants that there was an “unrushed and personalized rapport around the partner violence screening” [59, p. 962] and in general service-provider interactions. This participant elaborated that it was important for staff and/or providers to be

professional yet, um approachable…with your voice, and with eye contact, and just like your demeanor, like calm. Like, I’m not twitching my leg, or tapping my pencil. I have time for you to, like, be here. Really, like, physically and verbally show that you care, and you have time to listen to them, they’re important, and you want them to be safe, and get help. [59, p. 963]

Participants also spoke to needing more time to allow for deeper conversations and reflection, urging providers to “not just casually ask something and move on… [but get] a little deeper into it” [52, p. 10]. Participants wanted more time to build meaningful relationships and garner more impactful benefits from their experience [50,52,68]. As one participant commented:

I wish that it could have been more thorough and longer. Like if it became a permanent thing, I am sure that would fix the problem, but just the one a week and because it did have such a big impact on me, there wasn’t enough of it because now here I am, and now I’m on the other side of that, and there’s still a lot of work that needs to be done. [68, p. 12]

Another participant shared a desire for more frequent access to professional supports:

Yeah, I think they should offer us other counselors instead of the counselors that are here because they’re so busy–there’s so many people here that they can’t see us all and anytime if we have a crisis or are going through something, they’re booked out a week in advance, so what are we supposed to do? We have to just deal with it. [58, p. 9]

Participants also wished the duration of the programs lasted longer: “I wish it could last forever because there’s stuff that comes up now, you know, that I didn’t know” [50, p. 313].

Participants expressed the desire for more strengths focused discussion [47] and the importance of discussion with other IPV survivors to build support networks [55,67]. They wanted spaces “where women could talk and ask questions and share stories” [55, p. 163]. This desire was expressed for both in person and online formats. Some participants urged programs to better utilize technology for discussion and beyond:

Maybe doing telecommunication or skype videos. Cause I think that would have a greater impact...you could reach a lot more people. If they can’t get to you, they can literally just use their phone or computer and still talk to that person as long as they are in a safe space. And maybe they don’t feel comfortable [in the medical center] and they feel comfortable in their house talking. [68, p. 13]

Another participant agreed, stating:

I almost think it’s better over the phone because I am in my own comfortable space. I am not rushing to get into a hospital. Getting through traffic. I was resistant to doing therapy over the phone, but now, I have adapted to it, and I actually prefer it more. [68, p. 13]

### Implications for policy, practice, and research

This review highlights the value, impact, and limitations of trauma-informed programs addressing IPV, as experienced by participants. The range of program types—including interventions, housing models, assessment tools, training courses, and mobile apps—demonstrates the broad applicability of trauma-informed approaches. These programs came from both health care and IPV-specific services, underscoring how widely trauma-informed care has been adopted in both sectors [2,8,30,67].

Findings suggest that trauma-informed programs are most effective when they include education, skill development, opportunities for storytelling, and supportive networks. However, the rise of neoliberalism has undermined trauma-responsive services by prioritizing managerialism and system efficiency over relational care [68]. Trauma-informed approaches center what has happened to a person—not what is wrong with them—which often conflicts with biomedical and colonial models that focus on individual pathology.

Fully implementing trauma-informed principles requires a shift toward relational care that fosters trust and safety. This shift carries important implications for policy, practice, and research in systems aiming to adopt trauma-informed services.

### Implications for policy

The widespread satisfaction among IPV survivors with trauma-informed approaches highlights their value and calls on policymakers to allocate more resources toward their implementation. One key theme was the importance of time to build trust among participants and facilitators. The duration of interventions in this review varied widely—from one-time sessions]e.g., 47,52,59] to time-limited programs of 8–16 weeks e.g., [50,54,56–57,62–63,66], to long-term interventions spanning two years or more [e.g., 64,67], and live-in programs e.g., [49,58,61]. Some programs had broad internal variation: in Morales-Campos et al., participants attended for anywhere from two months to eight years [53], while in Dawson et al., attendance ranged from three to 20 sessions [48]. One program offered aftercare, but its uptake was unclear [63].

Naturally, such variation in duration likely influenced outcomes. Longer programs offered more time to foster trust, safety, collaboration, and peer support. At the same time, participants may have held different expectations based on the length of the intervention, making it difficult to assess how these timeframes shaped overall findings. Three studies explicitly recommended longer-term, consistent support. Participants asked for more sessions [64,68] and advocated against program cuts [50]. Others expressed a need for deeper conversations with providers about IPV and recommended that screening include more time and thoughtful questioning [52].

These requests reflect a desire for sustained, in-depth support, which in turn highlights the need for stable and adequate funding. The short-term grants that many front-line services rely on cannot provide the consistency needed for meaningful trauma-informed care. Policymakers must be willing to challenge current funding norms that prioritize efficiency over relational care.

Trauma-informed approaches demand a shift from service-centered to human-centered models [36]. As Armstrong [69] notes, significant resources are required to implement these approaches effectively—making policy support essential to ensure programs are truly trauma-informed.

### Implication for practice

This review centers survivors’ perspectives and offers direct guidance for practitioners aiming to design IPV programs that meet client needs [70]. Program satisfaction was consistently tied to the creation of trust, safety, and a supportive environment. While staying in a relationship where violence has occurred can complicate safety, standard practices—such as safety planning and offering alternative accommodations—can help foster a sense of agency and choice [71] These strategies, core to trauma-informed care, can guide practitioners in developing effective, client-centered programming.

Findings on program dissatisfaction further emphasize the importance of providing culturally relevant, accessible, and non-triggering services. Participant recommendations included incorporating strengths-based discussions, addressing immediate needs at intake, extending session length, using interactive formats, and incorporating technology. Consistent satisfaction across all studies offers reassurance that survivors value a wide range of IPV interventions.

Most programs included in the review were designed for women. Only one targeted male perpetrators, and four included both men and women. While gender-specific programming remains vital given the gendered nature of IPV, the lack of services for men and families signals a significant gap. Katreena Scott of the Centre for Research and Education on Violence Against Women and Children recently noted the limited availability of programming for perpetrators, often offered only after justice system involvement [72]. This is a missed opportunity, as childhood trauma is closely linked to later IPV perpetration. As Augusta Scott [73] states, “working with men who use violence involves both addressing the effects of trauma and learning to take responsibility for their use of violence” [p. 127].

Including children in programming is equally critical. Exposure to IPV negatively affects children’s mental health [74] and increases their risk of future victimization or perpetration [7,16]. As the National Center for Injury Prevention and Control notes, “a large body of evidence highlights the importance of intervening early to prevent future involvement in violence, including future risk of perpetrating partner violence” [75, p. 26]. Practitioners should explore ways to expand trauma-informed programming for families, children, and those who use violence.

Notably, most studies did not address all six SAMHSA trauma-informed principles, with ‘trustworthiness and transparency’ mentioned least often [27]. This suggests a need for more intentional implementation of the full framework—though it’s important to note that some programs may have used alternative definitions of trauma-informed care. Many studies also lacked clarity on how trauma-informed principles were applied, highlighting a broader issue: the need for researchers, organizations, and practitioners to explicitly define and articulate their trauma-informed strategies.

Effective implementation requires significant resources, knowledge, and a cultural shift within organizations [69]. Without this commitment, the term “trauma-informed” risks becoming an empty label. Confusing trauma-informed care with trauma-specific treatment further dilutes its meaning. Armstrong [69] cautions that progress may be overstated due to this conflation, while Ross et al. warn that the rise in popularity of the term has led to “standardized practices that are often depoliticized, degendered, medicalized, and individualized within systems subject to neoliberal constraints” [26, p. 2]. To ensure trauma-informed care remains meaningful and effective, it must be implemented intentionally—at the individual, organizational, and systemic levels—grounded in a structural and social justice lens.

### Implications for research

In addition to the practice need for clearly and intentionally implementing trauma-informed approaches, further research is essential to refine and compare how these approaches are applied in IPV settings. While this review used SAMHSA’s six principles, other frameworks exist [27]. For example, Wilson et al. outlined six IPV-specific trauma-informed principles: “establishing emotional safety, restoring choice and control, facilitating connection, supporting coping, responding to identity and context, and building strengths” [30, p. 586]. Future studies could explore which trauma-informed elements are most impactful and compare trauma-informed with non-trauma-informed interventions. It would also be valuable to assess how using multiple frameworks [e.g., trauma-informed and survivor-centered] affects service user experiences [61,68].

There is also a growing shift in literature from the term *trauma-informed* to *trauma and violence-informed*, which explicitly links trauma to structural, cultural, and systemic violence [Ross et al., 2023; McDonald et al., 2016]. This lens reframes the source of trauma as rooted not in individuals, but in broader injustices like racism and colonialism [Ross et al., 2023; Clark, 2016]. This is especially relevant to the gendered nature of IPV and highlights the intersection of interpersonal and systemic violence [McDonald et al., 2016]. However, only one included study used the term *trauma and violence-informed* [Jackson et al., 2020], and another used a *violence-informed* approach [Dawson et al., 2021]. Further research could explore whether this shift in terminology changes program delivery or participant experience. It may overlap significantly with SAMHSA’s principle of “cultural, historical, and gender issues,” but the practical implications of the shift remain unclear [27].

Barriers to accessing IPV programming were discussed in only three studies [Domoney et al., 2019; Schuler et al., 2011; Veale et al., 2020], often linked to stigma. Broader literature shows additional barriers such as shame, lack of awareness, and culturally inappropriate services [Colucci et al., 2013; McCleary-Sills et al., 2016; Petersen et al., 2005; Prentice et al., 2016]. For example, Domoney et al. [2019] found that some men “struggled with the language of abuse and perpetration” [p. 547]. The program responded by adjusting its intake language to encourage participation. More research is needed on barriers to service access, especially given IPV’s underreporting [Cullen, 2023] and how well-meaning policies may still miss survivor needs [Ryan et al., 2022].

While program satisfaction was more frequently reported than dissatisfaction or suggestions, this may reflect research design rather than actual experience. As Coyle [1999] observed, service users may hesitate to voice dissatisfaction due to cultural norms or fear of losing access to services. Future studies should intentionally create space for negative feedback and ensure participants feel safe to share. Understanding dissatisfaction is essential for improving care. Time is also a key factor in building the relational foundations of trauma-informed care. Eleven studies highlighted how trust—with providers, in groups, or within systems—contributed to program satisfaction. Program length varied widely, from single sessions to live-in, multi-year interventions. While no exact time requirement exists, healing from trauma is a long-term process. Menschner and Maul [76] suggest that 8–15 weekly sessions of 60–90 minutes are effective for adults with trauma or PTSD, though more research is needed to confirm optimal duration. These findings have important implications for policy and funding, supporting advocacy for adequate time and resources. Future research comparing programs with similar durations, but different approaches would be particularly useful.

Most studies relied on retrospective data collected months or even years after program completion. Valuable insights could be gained by interviewing participants both shortly after completing the program and again later to assess long-term impact. Trabold et al. for example, included participants who completed the program between 3 and 12 + months prior, offering a broader perspective [56]. Five studies collected data at multiple time points [49,55,60,64,66,], though Bouchard and Wong [63] were unable to analyze six-month follow-up data due to low response rates. Finally, while four studies were evaluations [63–66], none assessed how well trauma-informed principles were integrated. This represents a gap in the literature. More targeted research is needed to evaluate not just program outcomes, but how effectively trauma-informed frameworks are implemented in practice.

### Strengths & limitations

To our knowledge, this is the first scoping review to center participants’ experiences of trauma-informed IPV programs. This focus offers valuable insight for informing future policy and practice. Strengths of this review include its systematic approach and the involvement of multiple reviewers with relevant expertise throughout the review process. The review also applied a widely recognized definition of trauma-informed care [27] and accounted for variability in how programs implemented trauma-informed principles.

However, several limitations should be noted. Determining whether a program included at least one trauma-informed principle involved a degree of subjectivity, and other teams may have selected a different set of studies based on alternative interpretations or definitions of trauma-informed care. Choosing a different framework could have yielded different results. Moreover, only studies with qualitative data published in English were included, potentially excluding valuable quantitative findings or insights from non-English or gray literature. Optional community consultation was also not conducted, which might have enhanced validation and contextual depth.

The diversity of programs included presented additional challenges. Interventions varied widely in structure and duration, ranging from single-day sessions to multi-year, live-in programs, and were implemented over widely different time periods. This variability limited the ability to draw clear conclusions about program effectiveness and participant experience and also raises the possibility that longer interventions may be more likely to incorporate multiple trauma-informed principles. However, the data available did not allow us to systematically examine this relationship. Additionally, many studies lacked detailed information on the number of participants involved, the type of facility, and the specific context in which the intervention occurred, which limited our ability to assess how these factors influenced the integration and impact of trauma-informed principles.

A key limitation was the wide variation in both program duration and the timing of participant interviews. Some studies interviewed participants immediately after the intervention, while others collected data months or even years later. This inconsistency introduces potential recall bias and complicates comparisons across studies. The brevity of some interventions—particularly those lasting only a single day—may limit the opportunity to fully implement and observe the effects of trauma-informed principles.

Although all included studies incorporated at least one of SAMHSA’s six trauma-informed principles, none included all six. It remains unclear whether longer programs were more likely to incorporate a greater number of principles, or whether the number of principles present was associated with improved participant outcomes. Future research should explore whether program duration influences the depth and breadth of trauma-informed implementation, and whether more comprehensive inclusion of the six principles leads to greater participant benefit. Lastly, because this was a scoping review, the methodological quality of included studies was not assessed. As such, findings should be interpreted with caution and used to guide, rather than definitively determine, future research and practice.

## Conclusion

Exploring how individuals impacted by IPV experience trauma-informed programs and approaches is essential to providing care that meets their needs. This review was deliberate in highlighting the voices of service users and emphasizing their experiences. Our findings indicated that individuals from a wide variety of countries value programs that are trauma-informed. Contrary to current system-centred trends that trump efficiency and devalue relationality, this review highlighted the need for more relational approaches in service provision. We hope that these findings will encourage policymakers, practitioners, and researchers to engage in advocacy that supports the effective and sustained implementation of trauma-informed programming.

## Supporting information

S1 File PRISMA_2020_checklist_Aug 8. (DOCX)

S2 File Appendix A Extraction Table_Updated August 2024. (DOCX)

## References

[1] World Health Organization. Violence against women: Key facts. 2021.

[2] Baker L, Straatman AL, Etherington N, O’Neil B, Heron C, Sapardanis K. Towards a conceptual framework: Trauma, family violence and health. Knowledge Hub; 2016.

[3] Sardinha L, Maheu-Giroux M, Stöckl H, Meyer SR, García-Moreno C. Global, regional, and national prevalence estimates of physical or sexual, or both, intimate partner violence against women in 2018. Lancet. 2022;399(10327):803–13. doi: 10.1016/S0140-6736(21)02664-7 35182472 PMC8885817

[4] Tarshis S, Alaggia R, Logie CH. Intersectional and Trauma-Informed Approaches to Employment Services: Insights From Intimate Partner Violence (IPV) Service Providers. Violence Against Women. 2022;28(2):617–40. doi: 10.1177/1077801220988344 33591243

[5] Women and Gender Equality Canada. Gender-based violence glossary. Government of Canada; 2021.

[6] World Health Organization. Violence against women prevalence estimates, 2018. 2018.

[7] Cotter A. Intimate partner violence in Canada, 2018: An overview. Juristat. 2018.

[8] Drexler KA, Quist-Nelson J, Weil AB. Intimate partner violence and trauma-informed care in pregnancy. Am J Obstet Gynecol MFM. 2022;4(2):100542. doi: 10.1016/j.ajogmf.2021.100542 34864269

[9] Evans ML, Lindauer M, Farrell ME. A Pandemic within a Pandemic - Intimate Partner Violence during Covid-19. N Engl J Med. 2020;383(24):2302–4. doi: 10.1056/NEJMp2024046 32937063

[10] Gilbert LK, Zhang X, Basile KC, Breiding M, Kresnow M. Intimate partner violence and health conditions among U.S. adults. J Interpers Violence. 2023;38(1–2):237–61. 10.1177/08862605221080147 PMC9509488 35337195

[11] Gillum TL. African American survivors of intimate partner violence. J Aggress Maltreat Trauma. 2021;30(6):731–48.

[12] Hoffart R, Jones NA. Intimate partner violence and intergenerational trauma among indigenous women. International Criminal Justice Review. 2017;28(1):25–44. doi: 10.1177/1057567717719966

[13] Anyikwa VA. Trauma-Informed Approach to Survivors of Intimate Partner Violence. J Evid Inf Soc Work. 2016;13(5):484–91. doi: 10.1080/23761407.2016.1166824 27142906

[14] Trabold N, McMahon J, Alsobrooks S, Whitney S, Mittal M. A systematic review of intimate partner violence interventions. Trauma Violence Abuse. 2020;21(2):311–25. 29649966 10.1177/1524838018767934

[15] Herman J. Trauma and recovery. Basic Books; 2015.

[16] Hughes K, Bellis M, Hardcastle K, Sethi D, Butchart A, Mikton C, et al. The effect of multiple adverse childhood experiences on health. Lancet Public Health. 2017;2(8):e356–66. 10.1016/S2468-2667(17)30118-4 29253477

[17] Campbell JC. Health consequences of intimate partner violence. Lancet. 2002;359(9314):1331–6. doi: 10.1016/S0140-6736(02)08336-8 11965295

[18] Dillon G, Hussain R, Loxton D, Rahman S. Mental and physical health and intimate partner violence. Int J Fam Med. 2013. 10.1155/2013/313909 PMC3566605 23431441

[19] Loxton D, Dolja-Gore X, Anderson AE, Townsend N. Intimate partner violence adversely impacts health over 16 years. PLoS One. 2017;12(6):e0178138. doi: 10.1371/journal.pone.0178138 PMC5459340 28582406

[20] Wathen N. Health impacts of violent victimization on women and their children. Department of Justice Canada; 2012.

[21] Ross N, Morrison J, Cukier S, Smith T. Consuming carcinogens: women and alcohol. Our Chemical Selves. University of British Columbia Press. 2015. p. 188–227. doi: 10.59962/9780774828352-011

[22] Breiding MJ, Smith SG, Basile KC, Walters ML, Chen J, Merrick MT. National intimate partner and sexual violence survey, 2011. 2014. PMC4692457 25188037

[23] Hamberger L, Larsen S. Men’s and women’s experience of intimate partner violence. J Fam Violence. 2015;30(6):699–717.

[24] Elliott DE, Bjelajac P, Fallot RD, Markoff LS, Reed BG. Trauma-informed or trauma-denied. J Community Psychol. 2005;33(4):461–77.

[25] Levenson J. Trauma-Informed Social Work Practice. Soc Work. 2017;62(2):105–13. doi: 10.1093/sw/swx001 28339563

[26] Ross N, Brown C, Johnstone M. Beyond medicalized approaches to violence and trauma. J Soc Work. 2023.

[27] SAMHSA. SAMHSA’s concept of trauma and guidance for a trauma-informed approach. 2014.

[28] McDonald S, Thomson C, Fraser C, Morton-Bourgon K, Elliott K, McKinnon P. Victims of crime: Research digest. Department of Justice Canada; 2016.

[29] Perry B, Winfrey O. What happened to you? Flatiron Books; 2021.

[30] Wilson JM, Fauci JE, Goodman LA. Bringing trauma-informed practice to domestic violence programs. Am J Orthopsychiatry. 2015;85(6):586–99. 26594925 10.1037/ort0000098

[31] Morrissey JP, Jackson EW, Ellis AR, Amaro H, Brown VB, Najavits LM. Twelve-month outcomes of trauma-informed interventions. Psychiatr Serv. 2005;56(10):1213–22. 16215186 10.1176/appi.ps.56.10.1213

[32] Scheer JR, Poteat VP. Trauma-Informed Care and Health Among LGBTQ Intimate Partner Violence Survivors. J Interpers Violence. 2021;36(13–14):6670–92. doi: 10.1177/0886260518820688 30596315 PMC7023297

[33] Kulkarni S. Intersectional trauma-informed intimate partner violence services. J Fam Violence. 2019;34(1):55–64.

[34] Wathen CN, Schmitt B, MacGregor JCD. Measuring trauma- and violence-informed care. Trauma Violence Abuse. 2023;24(1):261–77. 34235986 10.1177/15248380211029399 PMC9660280

[35] Ryan C, Silvio D, Borden T, Ross NM. Pro-arrest and pro-charge policies as response to domestic violence. J Soc Work. 2022;22(1):211–38.

[36] Council of Parties. Journey to light: Final report of the Restorative Inquiry. Province of Nova Scotia; 2019.

[37] Pollock D, Peters MDJ, Khalil H, McInerney P, Alexander L, Tricco AC. Recommendations for scoping reviews. JBI Evidence Synthesis. 2022. 10.11124/JBIES-20-00167 33038124

[38] Arksey H, O’Malley L. Scoping studies. Int J Soc Res Methodol. 2005;8(1):19–32.

[39] Daudt HML, van Mossel C, Scott SJ. Enhancing scoping study methodology. BMC Med Res Methodol. 2013;13(1):1–9. 23522333 10.1186/1471-2288-13-48 PMC3614526

[40] Grant MJ, Booth A. A typology of reviews. Health Information & Libraries Journal. 2009;26(2):91–108. 19490148 10.1111/j.1471-1842.2009.00848.x

[41] McGowan J, Sampson M, Salzwedel DM, Cogo E, Foerster V, Lefebvre C. PRESS guideline statement. J Clin Epidemiol. 2016;75:40–6. 27005575 10.1016/j.jclinepi.2016.01.021

[42] Levac D, Colquhoun H, O’Brien KK. Scoping studies: advancing the methodology. Implement Sci. 2010;5:69. doi: 10.1186/1748-5908-5-69 20854677 PMC2954944

[43] Pollock D, Peters MDJ, Khalil H. JBI Evid Synth. 2022. 10.11124/JBIES-21-00416 35477565

[44] Peters MDJ, Marnie C, Tricco AC, Pollock D, Munn Z, Alexander L, et al. Updated methodological guidance for the conduct of scoping reviews. JBI Evid Synth. 2020;18(10):2119–26. doi: 10.11124/JBIES-20-00167 33038124

[45] Elo S, Kyngäs H. The qualitative content analysis process. J Adv Nurs. 2008;62(1):107–15. doi: 10.1111/j.1365-2648.2007.04569.x 18352969

[46] Page MJ, McKenzie JE, Bossuyt PM. The PRISMA 2020 statement. BMJ. 2021;372:n71. 10.1136/bmj.n71 PMC8005924 33782057

[47] Dagenhardt DMR, Mersky J, Topitzes JD, Schubert E, Krushas AE. Assessing polyvictimization. J Interpers Violence. 2022;37(19–20):NP17276–99. 10.1177/08862605211027998 34215168

[48] Dawson L, Einboden R, McCloughen A, Buus N. Open Dialogue in a women’s shelter. J Marital Fam Ther. 2021;47(1):136–49. 32990992 10.1111/jmft.12457

[49] Hetling A, Dunford A, Lin S, Michaelis E. Long-term housing and intimate partner violence. Affilia. 2018;33(4):526–42. doi: 10.1177/0886109918778064

[50] Jackson KT, Mantler T, Jackson B, Walsh EJ, Baer J, Parkinson S. Trauma-informed CBT following IPV. J Psychosom Obstet Gynecol. 2020;41(4):308–16. 10.1080/0167482X.2019.1707799 31902267

[51] Kahan D, Lamanna D, Rajakulendran T, Noble A, Stergiopoulos V. Trauma-informed intervention for homeless female survivors. Health Soc Care Community. 2020;28(3):823–32. 31814189 10.1111/hsc.12913

[52] Miller E, McCauley HL, Decker MR, Levenson R, Zelazny S, Jones KA. Reproductive coercion intervention implementation. Perspect Sex Reprod Health. 2017;49(2):85–93. 28272840 10.1363/psrh.12021 PMC5453817

[53] Morales-Campos DY, Casillas M, McCurdy SA. Support group for Hispanic women living with gender-based violence. J Immigr Minor Health. 2009;11:57–65. 18561024 10.1007/s10903-008-9153-3

[54] Reid N, Kron A, Rajakulendran T, Kahan D, Noble A, Stergiopoulos V. Promoting wellness and recovery. Violence Against Women. 2021;27(9):1297–316. 32573362 10.1177/1077801220923748

[55] Schuler SR, Trang QT, Ha VS, Anh HT. Engaging abused women and communities in Vietnam. Violence Against Women. 2011;17(11):1421–41. 22240404 10.1177/1077801211433990

[56] Trabold N, O’Malley A, Rizzo L, Russell E. A gateway to healing. J Community Psychol. 2018;46(4):418–28.

[57] Veale A, Shanahan F, Hijazi A, Osman Z. Engaging men among Syrian refugees. Intervention. 2020;18(1):52–60.

[58] Wood L, Clark D, Heffron LC, Schrag RV. Survivor-centered advocacy. Adv Soc Work. 2020;20(1):1–21.

[59] Decker MR, Flessa S, Pillai RV, Dick RN, Quam J, Cheng D. Trauma-informed partner violence assessment. J Womens Health. 2017;26(9):957–65. 10.1089/jwh.2016.6093 28375750

[60] Shayani DR, Danitz SB, Low SK, Hamilton AB, Iverson KM. Comparative thematic analysis of counseling interventions. Int J Environ Res Public Health. 2022;19(5):2513. 35270204 10.3390/ijerph19052513 PMC8909494

[61] Wood L, McGiffert M, Fusco RA, Kulkarni S. Transitional housing impact. Child Adolesc Soc Work J. 2022. 10.1007/s10560-021-00809-1 PMC8785383 35095183

[62] Woollett N, Bandeira M, Hatcher A. Trauma-informed art and play therapy. Child Abuse Negl. 2020;107. 10.1016/j.chiabu.2020.104564 PMC7494566 32512265

[63] Bouchard J, Wong JS. Disparate approaches to IPV intervention. Deviant Behav. 2021;42(11):1396–415.

[64] Domoney J, Fulton E, Stanley N, McIntyre A, Heslin M, Byford S. For Baby’s Sake intervention. J Fam Violence. 2019;34(6):539–51.

[65] Ragavan MI, Ferre V, Bair-Merritt M. Thrive mobile application. Health Promot Pract. 2020;21(2):160–4. 31874566 10.1177/1524839919890870

[66] Shai N, Pradhan GD, Shrestha R, Adhikari A, Chirwa E, Kerr-Wilson A. PLoS One. 2020;15(5):e0232256. 10.1371/journal.pone.0232256 PMC7237029 32427999

[67] Cronholm PF, Dichter ME. Systems of care and trauma-informed approach. Am Fam Physician. 2018;97(11). 30215942

[68] Brown C, Ross N, Johnstone M. Challenging neoliberalism and biomedicalism. Qual Health Res. 2022;32(5). 10.1177/10497323211069681 35382646

[69] Armstrong EM. Promise and pitfalls. J Fam Violence. 2023;38:841–53.

[70] Langhinrichsen-Rohling J, Capaldi DM. Developing prevention programs for IPV. Prev Sci. 2012;13(4):410–4. 22752380 10.1007/s11121-012-0310-5 PMC3405177

[71] Cottrell B. Making changes. Nova Scotia Advisory Council on the Status of Women; 2022.

[72] Canadian Press. Ontario needs more programs for men to end domestic abuse. 2024.

[73] Scott T. Exploring trauma and masculinity. In: Brown C, MacDonald J, editors. Critical Social Work. 2020.

[74] Wathen CN, MacMillan HL. Children’s exposure to intimate partner violence. Paediatr Child Health. 2013;18(8):419–22. 24426794 PMC3887080

[75] Niolon PH, Kearns M, Dills J, Rambo K, Irving S, Armstead TL. Preventing intimate partner violence across the lifespan. CDC; 2017.

[76] Menschner C, Maul A. Key ingredients for successful trauma-informed care implementation. Center for Health Care Strategies; 2016. https://www.chcs.org/resource/key-ingredients-for-successful-trauma-informed-care-implementation/

