# Supplementary material for: How survivors of intimate partner violence experience trauma-informed care: A scoping review
Source: PLoS One. 2026 Apr 22;21(4):e0334623. doi: 10.1371/journal.pone.0334623 (PMC13102228; doi:10.1371/journal.pone.0334623)
Supplement: S2 File — (DOCX) [file pone.0334623.s002.docx]

Appendix A

*Summary of Included Studies*

| **First author, year** | **Title** | **Aim of study** | **Method** | **Participants** | **Location** | **IPV trauma informed program/approach** | **Program Duration** | **Trauma Informed Principles** |
| --- | --- | --- | --- | --- | --- | --- | --- | --- |
| Bouchard, 2020 | Disparate approaches to intimate partner violence intervention: A preliminary investigation of participant outcomes across two community based programs | To comparatively evaluate the effectiveness of two intervention programs (Help Yourself to Healthy Relationships [HYHR] and Managing Stress and Personal Relationships [MSPR]) targeted towards male perpetrators of intimate partner violence (IPV). | Mixed methods comparative program evaluation; single-group pretest posttest design with additional 6 month follow up survey; survey used validated instrument to assess abusive behaviors; pretest included questions about participant’s history and demographics; posttest included two open-ended questions assessing participant feedback about program content and knowledge gains and four close-ended question assessing satisfaction with program; textual analysis used for analysis of qualitative program outcomes.  *Programs part of a larger simultaneous evaluation of 12 IPV intervention programs operating in British Columbia. | Male perpetrators of IPV who completed the MSPR or HYHR program.  29 MSPR participants from five program cycles (20 provided at least one qualitative response and 17 completed post-test) and 23 HYHR program participants from four program cycles (19 provided at least one qualitative response and 15 completed post-test).  MSPR: Ages 25-74, mean age 41.27. Ethnicity: 52% Caucasian, 30% Persian, 13% other, 4% First Nations. 52% Born in Canada. Martial Status: 43% married, 30% living common-law, 22% separated, 1% single. 87% have children and 70% have children living at home with participant.  HYHR: Ages 20-66, mean age 40.43. Ethnicity: 55% Caucasian, 35% Other, 17% South Asian, 3% First Nations. 72% Born in Canada. Marital Status: 31% married, 8% single, 17% living common law; 14% divorced, 10% separated. 72% have children and 62% have children living at home with participant. | British Columbia, Canada | HYHR: a group feminist/trauma-informed program. Participants are screened for readiness. Sessions are facilitated by a male/female team with experience working with family violence and are delivered in English or Farsi. Each group session includes a check-in, topic introduction, group discussion and check-out; one session is focused on stress and trauma.  MSPR: a group cognitive behavior therapy (CBT) oriented anger/stress management program. Participants are assessed for risk level and needs before beginning the group. Groups are facilitated by a male/female team with training and experience working with men who have been abusive in relationships and are facilitated in English or Punjabi. Sessions include discussion, videos, role plays, and activities; one session is focused on the impact of substance abuse, trauma, and mental health. | HYHR: 12 2-hour weekly group sessions with ongoing group and one-on-one monthly aftercare sessions offered following the program. MSPR: 12 weekly group sessions that are 3 hours in duration. Weekly drop-in, group-based, peer-led aftercare sessions are offered at the end of the program. | Safety; peer support; cultural, historical, and gender issues |
| Dagenhardt, 2021 | Assessing polyvictimization in a family justice center: Lessons learned from a demonstration project | To examine the experience of administration of the Polyvictimization Assessment Tool (PAT) from staff and client perspectives in order to inform future tool developments and assessment procedures. | Qualitative study; interviews and focus groups; grounded theory. | 4 Family Justice Centre (FJC) staff who administered the PAT and 10 clients who completed the initial or revised version of the PAT.  Staff: All women of varying racial/ethnic backgrounds.  Clients: All women of varying racial/ethnic and socioeconomic backgrounds. | Urban area of the upper Midwest, United States of America | The Polyvictimization Assessment Tool (PAT), an assessment tool that is administered conversationally by staff and measures the experience of potentially traumatic incidents, adverse events and conditions, and trauma symptoms. It was developed and tested at one of the U.S’ largest and most comprehensive FJCs providing co-located multi-sector responses to domestic violence. The PAT was administered by four client advocates (FJC staff members) who established ongoing relationships with participants. The PAT began with advocates describing the purpose and voluntary nature of the assessment. Staff used the information to suggest services and referrals to clients. | 1-time assessment that could be completed over multiple sessions if needed. | Safety; trustworthiness and transparency; empowerment, voice and choice; cultural, historical, and gender issues |
| Dawson, 2021 | Beyond polyphony: Open dialogue in a women's shelter in Australia as a possibility for supporting violence-informed practice | To explore the experience and impact of Open Dialogue network meetings at an Australian women’s community-based shelter from the perspective of service users. | Qualitative study; semi-structured interviews; thematic analysis. | 6 women who attended the Australian shelter and participated in at least one open dialogue meeting; 7 open dialogue practitioners who worked or volunteered at the shelter and had completed open dialogue training and received ongoing supervision.  Shelter participants: people who identified as women from a variety of backgrounds. Often women at the shelter come from historically disadvantaged groups.  Open dialogue practitioners: two shelter case managers, shelter general manager, two mental health nurse academics, one clinical psychologist, one registered nurse. | Metropolitan area of Australia | Open dialogue, a family/social network-centered psychotherapeutic approach to responding to people in crisis and distress. The network meetings are characterized by the dialogical approaches of deep, appreciative listening, open-ended and relational/circular questioning, and the use of practitioners’ reflections in front of the person of concern and the network members.  Open dialogue meetings were led by a pair of Open Dialogue therapists or shelter staff or volunteers who had completed introductory Open Dialogue training. Service users were encouraged to invite members of their network to meetings. | Open dialogue meetings were 1.5 hours long, offered every 2 weeks. Each participant had attended at least 1 meeting, with some participants being described as having attended more than 3, 10, or 20 meetings. | Safety; trustworthiness and transparency; peer support; collaboration and mutuality; cultural, historical, and gender issues |
| Decker, 2017 | Implementing trauma-informed partner violence assessment in family planning clinics | To evaluate the uptake and impact of a brief, trauma-informed, universal IPV/RC assessment and education intervention. | Mixed-methods study; quasi-experimental, single group pretest-posttest study and in-depth qualitative interviews; thematic analysis. | Qualitative interviews: 26 patients and nine providers at two participating family planning health centers.  Quantitative sample: 132 English-speaking women patients ages 18-35; 68 patients completed the post-test. Race: 50.8% Black or African American, 37.9% White, and 11.4% Other. 93% born in the US. Relationship status: 57.6% dating one person/in a serious relationship, 34.9% single/dating more than one person, 7.6% married. 37% had experienced physical or sexual IPV. | Baltimore, Maryland, United States of America | ARCHES intervention, implemented through Project Connects in two family planning programs in greater Baltimore, Maryland. The intervention utilizes provider discussion and palm-sized safety cards to provide enhanced universal assessment, harm reduction counselling, and offering to connect patients with domestic violence advocates in real time. Providers received trauma informed care training. | Brief (3-5 minute) intervention. | Safety |
| Domoney, 2019 | *For Baby’s Sake*: Intervention development and evaluation design of a whole-family perinatal intervention to break the cycle of domestic abuse | To summarise the process of developing For Baby’s Sake and describe the evaluation design using early data to illustrate successes and challenges. | Mixed-methods evaluation; face-to-face research interviews at three time points (program sign-up, 1 year, and 2 years) that included self-report and researcher-administered questionnaires on domestic abuse, mental health, and parenting and infant outcomes; a qualitative interview; and observed measures of child development and parenting. | 40 program participants from families assessed as currently experiencing domestic abuse: 27 women and 13 men.  Women: Mean age of 28, 75% White British, 85% completed education beyond age 16, 37% married or co-habiting, 40% first time parents.  Men: Mean age of 29, 85% White British, 55% completed education beyond age 16, 54% married or co-habiting, 38% first time parents. | England | For Baby’s Sake, a trauma-informed, attachment-based, strengths-based, whole-family intervention that works to break cycles of domestic abuse and improve outcomes for children. Works with both parents regardless of if they plan to stay together as a couple. Staff delivering the program complete a significant amount of training and come from a variety of professional backgrounds, such as police, probation, the domestic violence sector, and early years’ services. The intervention was developed as a structured, modular program which is delivered flexibly to meet individual needs. Therapeutic sessions are delivered face-to-face and utilize a range of therapeutic techniques to support behaviour change and recovery from trauma, with Inner Child work at the therapeutic core of the program. Observation and video tools are used for therapeutic and attachment-based parenting work. | Up to 2.5 years - staff work with parents from pregnancy to 2 years postpartum. | Safety; collaboration and mutuality; empowerment, voice and choice |
| Hetling, 2018 | Long-term housing and intimate partner violence: Journeys to healing | To examine how housing helps residents heal from trauma, understand how women conceptualize living and participating in supportive programs at Dina’s Dwellings, and offer suggestions to ensure Dina’s Dwellings is a safe, supportive, and positive space for residents. | Qualitative study; In-depth qualitative case study; longitudinal; focus groups and in-depth interviews; feminist grounded theory. | 8 residents of Dina’s Dwellings . All women, ages early 20s to early 50s, ethnicities included Caucasian, African American, Latina, and South Asian, all qualified for low-income housing and had documented mental health disabilities. | New Brunswick, New Jersey, United States of America | Dina’s Dwellings, affordable permanent housing for women survivors of IPV and their children. The building has 10 apartments and a community space. Supportive services and referrals are also provided to residents. | Long-term; research began about 5 months after most women had moved into the building. | Safety; empowerment, voice and choice |
| Jackson, 2020 | Exploring mothers’ experiences of trauma and violence-informed cognitive behavioural therapy following intimate partner violence: A qualitative case analysis | To determine the perceived value and acceptability of trauma and violence-informed cognitive behavioral therapy (TVICBT) to support maternal mental health and maternal-infant attachment. | Qualitative study; semi-structured interviews; inductive content analysis.  *Part of a larger mixed-methods study called Promoting Attachment Through Healing (PATH). | 3 Caucasian postpartum women who had experienced IPV and who had completed an eight-week TVICBT intervention. | Southwestern Ontario, Canada | TVICBT, a combination of Cognitive Behavioural Therapy (CBT) and trauma and violence informed care (TVIC). TVICBT occurs within a usual CBT session structure and is a problem solving process between the health care provider and woman within the context of TVIC. The TVICBT intervention treated IPV-related depression, anxiety, and/or PTSD symptomatology and was facilitated by a Perinatal Clinical Nurse Specialist. | 8-week intervention. | Safety; collaboration and mutuality |
| Kahan, 2020 | Implementing a trauma-informed intervention for homeless female survivors of gender-based violence: Lessons learned in a large Canadian urban centre | To examine service user and provider experiences of a trauma-informed, peer-facilitated group psychosocial intervention (Peer Education and Connection through Empowerment [PEACE]) targeting female identified youth experiencing homelessness and gender-based violence. | Qualitative study; semi-structured interviews; thematic analysis. | 12 service users who had participated in the PEACE program at Covenant House. and 7 additional stakeholders (direct program administrators, Covenant House staff, and peer mentors).  Service users: 11 female-identified and one bi-gender, between 19 and 24 years old, most born in Canada, included Caucasian, Black, South Asian and participants of mixed ethnic background. | Toronto, Canada | PEACE, a peer supported and trauma-informed group intervention which aims to support and empower young female-identified survivors of gender-based violence experiencing homelessness. Group activities focus on health promotion and a variety of relevant psycho-educational topics; groups follow a standardized curriculum with room for flexibility based on participant-identified needs and preferences. Each group has approximately eight participants in order to allow for privacy and emotional safety. Each group is facilitated by a health promotion coordinator and two trained and paid peer mentors. PEACE launched in 2017 at Covenant House Toronto, Canada's largest agency serving youth who have experienced or are at-risk of homelessness. | Approximately 3 months duration. | Safety; trustworthiness and transparency; peer support; collaboration and mutuality; empowerment, voice and choice; cultural, historical, and gender issues |
| Miller, 2017 | Implementation of a family planning clinic–based partner violence and reproductive coercion Intervention: provider and patient perspectives | To explore the acceptability of the ARCHES (Addressing Reproductive Coercion in Health Settings) intervention and barriers to implementation in Pennsylvania family planning clinics. | Qualitative study; semi-structured interviews; consensus and open coding.  *Part of a larger cluster randomized controlled trial of the ARCHES intervention. | 18 providers and 5 administrators who had received ARCHES training as well as 49 patients who reported a history of partner violence. All participants were from 11 family planning clinics in Pennsylvania.  Providers/administrators: All women, almost two-thirds had been working in reproductive health care for more than 10 years.  Patients: All women. 33% 18–21 years old, 39% 22–26 and 29% 27–30. Racial identity: 70% White, 20% African American or Black, 10% multiracial or other. | Pennsylvania, United States of America | ARCHES, a trauma-informed intervention addressing IPV and reproductive coercion provided to all women seeking care, regardless of exposure to violence. The intervention seeks to educate women about available resources and harm reduction strategies and increase patient and provider comfort in discussing reproductive coercion and related abuse. Providers give all patients two educational cards about relationship violence and discuss it with them (the second card is to give to a friend or family member). The card provides a way for patients to obtain information without disclosing abuse, empowers them to share information with others, and includes harm reduction information. Providers also support referrals to victim services as needed. | Brief intervention. | Safety; peer support; empowerment, voice and choice |
| Morales-Campos, 2009 | From isolation to connection: Understanding a support group for Hispanic women living with gender-based violence in Houston, Texas | To understand Hispanic women’s experience of participating in a support group to prevent gender-based violence (GBV). | Qualitative study; internal documents, participant observation, and in-depth interviews; consensus coding and code networks. | 30 women who had sought assistance for violence or abuse at La Rosa Family Services. 90% Mexican/Mexican American, remainder Central American, South American, or other. Age range 25 to 71, average age 41. Marital status: 50% married, 20% divorced, 6.7% common-law, 3.3% single. Immigration status: 43% U.S. citizens, 30% residents, 27% undocumented immigrants, resided in the U.S between 3 and 46 years (mean 37 years). | Houston, Texas, United States of America | Support group of Hispanic women exposed to GBV and/or abuse. Facilitated by professionals at La Rosa Family Services, a community-based organization in Houston. Approximately 20–25 women attend each support group during a week and it is a free service. | Weekly support group, interviewees had first attended the support group 2 months to 8 years ago. | Peer support; empowerment, voice and choice; cultural, historical, and gender issues |
| Ragavan, 2020 | Thrive: A novel health education mobile application for mothers who have experienced intimate partner violence | To conduct a formative evaluation of the content, design, safety features, and applicability of the Thrive smartphone app. | Formative evaluation; structured interviews and a rating scale; inductive thematic analysis. | 16 providers (4 social workers, 3 IPV advocates, 9 health care providers) and 8 IPV survivors. | United States of America | Thrive, a trauma-informed, user-friendly Smartphone based mobile app created to address the unmet health needs and improve the well-being of mothers who have experienced IPV. Thrive contains three sections: Myself (maternal self-care, coping skills, and trauma-informed yoga); My Child (reducing childhood stress, promoting mother–child communication, and talking to children about IPV); and My Life (resources including housing, education, child care, IPV agencies, and national and state IPV and parenting hotlines). Thrive contains a variety of media (text, audio, video) as well as links to videos, websites, and other apps. | Participants were instructed to use the Thrive app for 10-20 minutes. | Collaboration and mutuality; cultural, historical, and gender issues |
| Reid, 2021 | Promoting wellness and recovery of young women experiencing gender-based violence and homelessness: The role of trauma-informed health promotion interventions | To explore the experiences of young women experiencing gender-based violence (GBV) and homelessness who participated in a community-based, trauma-informed group intervention in Toronto, Canada. | Qualitative study; semi-structured interviews; thematic content analysis. | 18 women experiencing homelessness who were enrolled in The Peer Education and Connection through Empowerment program. Aged 16-24 years. Ethnic identity: 33% Black, 22% Mixed, 17% Caucasian, 17% Asian, 11% other. Education level: 50% high school or less, 39% some. secondary school, 11% completed secondary school. Employment status: 39% unemployed, 33% student, 28% employed, 17% volunteer. | Toronto, Canada | The Peer Education and Connection through Empowerment program, a community- and group-based health promotion intervention using a trauma informed care framework. The intervention is offered at Covenant House in Toronto; cohorts of eight people participate in psychoeducation (identity formation, self-image, women’s health, healthy relationships, coping mechanisms, and leadership) and social activities (yoga, arts and crafts, and meal preparation). Participants receive support and mentorship from a Health Promotion Coordinator and two trained and remunerated peer mentors with lived experience who assist in group cofacilitation. Participants are supported and encouraged to actively engage in discussions and contribute ideas for curricula based on their needs and preferences. | Intervention is offered 2 hours weekly over 16 weeks. | Safety; peer support; collaboration and mutuality; empowerment, voice and choice |
| Schuler, 2011 | Qualitative study of an operations research project to engage abused women, health providers, and communities in responding to gender-based violence in Vietnam | To establish the extent to which a community project addressing gender-based violence (GBV) in Vietnam was empowering for abused women and to refine program design, provide process documentation, and provide qualitative evidence of the project’s impact from the perspectives of various participants and stakeholders. | Qualitative study; in-depth ethnographic interviews and focus groups; grounded theory and narrative analysis. Data was collected during four field visits over time.  *Evaluates and describes an action research project. | Interviews were conducted with 58 married women (35 of whom had experienced GBV), 29 married men, 29 key informants, 15 health service providers. In addition, there were 15 focus group discussions (6 with women, 4 with men, and 5 with key informants). Participants were from two (out of seven) communes in Vietnam where the project was implemented. | Cua Lo, Nghe An Province, Vietnam | “An Integrated Model for Gender-Based Violence Prevention in Community and Clinic Settings”, developed through community workshops. The model includes sensitization training for professionals; awareness raising in the community; training for health providers; establishing a hospital-based counselling centre; developing community-based support systems for abused women; community and policy level advocacy; and documentation, evaluation, and dissemination efforts. | Research spanned 28 months, project was implemented for at least this long. | Collaboration and mutuality; cultural, historical, and gender issues |
| Shai, 2020 | “I got courage from knowing that even a daughter-in-law can earn her living”: Mixed methods evaluation of a family-centred intervention to prevent violence against women and girls in Nepal | To assess the effectiveness of the Sammanit Jeevan intervention conducted in two migrant communities in Baglung district, Nepal. | Evaluation; modified interrupted time series study and in-depth interviews; thematic analysis. | Qualitative interviews: 6 young married women, 4 older women (mothers-in-law), 4 young married men and 2 older men (fathers-in-law).  Quantitative sample: 200 women (100 young married women and 100 mothers-in-law) and 157 men (78 husbands and 79 fathers-in-law).  Young women: Age: 47% 16-24 years old, 29% 25-34 years old, 23% 35-44 years old, 1% 45-54 years old. Ethnicity: 44% Janajati, 29% Chhetri, 14% Dalit, 12% Brahmin, 1% Other. Education level: 14% none, 11% primary, 53% secondary, 22% above SEC. 99% currently married, 3% in polygamy, 26% married to a relative, 1% migrated for work.  Older women: Age: 1% 25-34 years old, 8% 35-44 years old, 30% 45-54 years old, 61% over 55 years. Ethnicity: 45% Janajati, 27% Chhetri, 13% Dalit, 13% Brahmin, 2% Other. Education level: 92% none, 7% primary, 0% secondary, 1% above SEC. 87% currently married, 28% in polygamy, 19% married to a relative, 0% migrated for work.  Young Men: Age: 20.5% 16-24 years old, 50% 25-34 years old, 21.8% 35-44 years old, 7.7% 45-54 years old. Ethnicity: 44.9% Janajati, 32.1% Chhetri, 11.5 % Dalit, 11.5% Brahmin. Education level: 1.3% none, 9% primary, 56.4% secondary, 33.3% above SEC. 94.4% currently married, 0% in polygamy, 16.7% married to a relative, 71.8% migrated for work.  Older Men: Age: 1.3% 25-34 years old, 5.1% 35-44 years old, 20.3% 45-54 years old, 73.4% over 55 years. Ethnicity: 45.6% Janajati, 29.1% Chhetri, 11.4% Dalit, 12.7% Brahmin, 1.3% Other. Education level: 50.6% none, 31.7% primary, 13.9% secondary, 3.8% above SEC. 97.5% currently married, 8.9% in polygamy, 20.3% married to a relative, 65.8% migrated for work. | Rural Baglung district, Nepal | Sammanit Jeevan (Living with Dignity), a participatory, group based, and family-centred model to reduce IPV, change harmful gender and social norms, and improve young married women’s economic conditions through women-led income generating activities (IGAs). Sessions were focused on transforming gender norms, economic empowerment, and IGA skill building and development; sessions were conducted in separate same age and sex groups, with all groups meeting together for discussions every three sessions. Sessions were co-facilitated by one male and one female facilitator. Each family receives in-kind (materials or live-stock) start-up funding equivalent to $150 to start a women-led IGA. | 20 weekly 3-hour sessions. | Peer support; empowerment, voice and choice; cultural, historical, and gender issues |
| Shayani, 2022 | Women tell all: A comparative thematic analysis of women’s perspectives on two brief counseling interventions for intimate partner violence | To assess the acceptability and usefulness of two interventions: Recovering from IPV through Strengths and Empowerment (RISE) and Enhanced Care as Usual (ECAU). | Mixed-methods randomized clinical trial; semi-structured interviews and quantitative helpfulness ratings; hybrid deductive-inductive analytic approach.  *Part of a larger randomized clinical trial, primary quantitative findings are described elsewhere. | 58 female patients of the Veterans Health Administration: 28 in RISE and 30 in ECAU (average age 39.21).  RISE: Mean age 28; Race/Ethnicity: 96.4% White Non-Hispanic, 67.9% White/Caucasian, 25% Black, 3.6% Asian, 3.6% Multiple Races, 3.6% Non-white/Hispanic. Sexual orientation: 64.3% Heterosexual, 10.7% Lesbian/Gay, 17.9% Bisexual, 7.1% Pansexual. Relationship status: 25% Married/Cohabitating, 25% Living Together/Not Married, 10.7% Not Living Together/Not Married, 25% Single, 7.1% Separated, 7.1% Other. 28.6% earn $75,000 or more. 57.1% Work Full Time, 50% Some College/Associate education.  ECAU: Mean age 30; Race/Ethnicity: 83.3% White Non-Hispanic, 53.3% White/Caucasian, 16.7% Black, 6.7% Asian, 13.3% Multiple Races, 16.7% Non-white/Hispanic. Sexual orientation: 80% Heterosexual, 3.3% Lesbian/Gay, 13.3% Bisexual, 3.3% Pansexual. Relationship status: 16.7% Married/Cohabitating, 16.7% Living Together/Not Married, 3.3% Not Living Together/Not Married, 26.7% Single, 30% Separated, 6.7% Other. 16.7% earn $75,000 or more. 27.6% Work Full Time, 53.3% Some College/Associate education. | New England, United States of America | RISE, a brief, clinician-administered, variable-length, modular, individualized psychosocial counseling intervention developed for women experiencing IPV. Participants choose how many sessions to complete (up to the 6 session limit) and which module they want to focus on in each session. Sessions consist of a safety check-in, self-efficacy tracking, goal review, and module selection (each module includes specific handouts, exercises, and related goal setting). The six-modules are: (A) Safety Planning, (B) Education on Health Effects of IPV and Warning Signs, (C) Improving Coping and Self-Care, (D) Enhancing Social Support, (E) Making Difficult Decisions, and (F) Connecting with Resources and Moving Forward.  ECAU, an advocacy and best practice-based intervention. Participants received an educational brochure to provide psychoeducation, then are provided information on local and national resources and safety planning, and can optionally engage in safety planning with the provider. | RISE: 1-6 sessions.  ECAU: One-time, 60-min intervention. | Empowerment, voice and choice |
| Trabold, 2018 | A gateway to healing: A community-based brief intervention for victims of violence | To explore key features and self-reported changes associated with a community-based, trauma-informed brief intervention (TIBI) for victims of IPV. | Qualitative study; semi-structured interviews; content analysis and cross-case analysis. | 15 women who have experienced IPV and completed TIBI in the last three years. Age range 26-56, average age 42.2. Race: 86.7% White, 6.7% Black, 6.7% Biracial. Relationship status: 66.7% separated from partner, 26.7% in relationship, 20% separated during intervention, 6.7% contemplated reconciliation. | Northeastern United States of America | TIBI, a community-based, individually focused, trauma informed, strengths-based brief therapy model of care. It is based on social cognitive theory and was developed by a group of women with vast backgrounds, including an IPV survivor and clinicians. It is delivered by clinicians with mater's level counseling degrees and extensive trauma training. Sessions cover psychoeducation, distress tolerance and relaxation, cognitive restructuring, safety planning and trauma trigger management, decision making, and healthy relationships. | Delivered over 10 sessions (45-60 minutes each). Delivered every 1-2 weeks for the first 6 weeks and every 3-4 weeks thereafter. | Safety; trustworthiness and transparency; collaboration and mutuality; empowerment, voice and choice |
| Veale, 2020 | Engaging men to promote resilient communities among Syrian refugees in Lebanon | To examine the impact of the Engaging Men intervention on Syrian refugees and Lebanese men. | Qualitative study; focus groups, family visits, and interviews; thematic analysis. | Focus groups: 130 men who had completed the Engaging Men training programme in Lebanon in the last year (80% Syrian refugees and 20% Lebanese), 28 wives of male participants, and 17 children of male participants (11 sons and 6 daughters). Additionally: 10 family visits (researchers met with former participants and their wives separately) and 10 individual interviews with former participants conducted by peer researchers. | Lebanon | Engaging Men intervention, a group training course with four key objectives: promoting peaceful interactions with others, reducing violence and gender-based violence, child protection and caregiving, and increasing community safety and harmony through a community project. Facilitators led group dialogue and adapted the programme to suit the cultural context (for example, using verses from the Koran, sitting in a circle, and not using a flip chart to accommodate for varying levels of literacy). Session topics were: resilient families and communities and the importance of men; understanding gender roles; gender relations; gender roles in action; the cycle of violence; violence against women; non-violence communication; men as nurturers; stress and human development; the impact of care giving; protecting children; men and women as agents of change. The programme was designed to include planning and implementing a community protection project, but in many cases the funds were used to buy household items that addressed the protection needs of families instead. | 12-week training course. | Trustworthiness and transparency; peer support; empowerment, voice and choice; cultural, historical, and gender issues |
| Wood, 2020 | Voluntary, survivor-centered advocacy in domestic violence agencies | To understand how to best facilitate survivor-centered approaches in a voluntary service format from the perspective of survivors. | Qualitative study; semi-structured interviews; thematic analysis.  *Part of a larger study considering the experiences of services for DV survivors using shelter and transitional housing programs. | 25 female-identified survivors dwelling in DV emergency shelter (60%) and transitional housing programs (40%). Age range 25-56, average age 37.5. Racial/ethnic background: 44% White, 32% African American, 16% Latinx, 8% Multiracial. Education: 28% some high school, 32% high school/GED, 32% some college, 8% college graduate. | Midwestern state and large Southwestern state, United States of America | Voluntary survivor-centered advocacy, a service model based on a feminist empowerment approach to promote individualized survivor well-being. Survivor centered advocacy acknowledges the variety of needs of individual survivors and is sensitive to the diverse needs of survivors and their families. The four participating domestic-violence focused organizations provided counseling and supportive services, shelter and other housing programs, and advocacy services for survivors. The participating organizations all identified as using a voluntary service model. | Live-in programs, duration not provided. | Safety; collaboration and mutuality; empowerment, voice and choice; cultural, historical, and gender issues |
| Wood, 2022 | “The propellers of my life” The impact of domestic violence transitional housing on parents and children | To understand the impact of DV program provided transitional housing (DVTH,) especially on child and parenting related needs and outcomes. | Mixed-methods study (only qualitative data reported); multiple structured interviews; thematic analysis.  *Data from a broader process evaluation. | 27 female domestic violence (DV) survivors with children living in a DVTH site. Ages: 41% 25-34 years old, 37% 34-44 years old, 22% 45 years or older. Primary language: 67% English, 33% Spanish. Race/ethnicity: 48% Latinx, 30% other, 22% Black. Education: 52% Middle school/some high school, 22% any college, 26% currently in school. | Southwestern United States of America | DVTH, a program that provides apartments, primarily clustered units, with a few scattered units, and voluntary supportive services addressing trauma, safety, economic barriers, and basic needs. This program is part of a larger DV focused agency serving survivors in a large urban county of the US. The DVTH program is over 20 years old and serves low-income and homeless adults and families who have experienced interpersonal violence. | 12-month program, with option to extend up to 24 months depending on the needs of the participant. | Safety; empowerment, voice and choice |
| Woollett, 2020 | Trauma-informed art and play therapy: Pilot study outcomes for children and mothers in domestic violence shelters in the United States and South Africa | To explore effects of a pilot intervention combining trauma focused cognitive behavior therapy (TF-CBT) with art and play therapy and to compare efficacy between high and low-middle income contexts (New York City and Johannesburg, South Africa). | Mixed-methods study; standardized measures and semi-structured interviews; thematic coding. | 21 children and 16 mothers living in domestic violence shelters.  Children: 71% from New York City and 29% from Johannesburg. Age: between 5-14 years old, mean 9.5 years old. 52% female, 48% male. | New York City, United States of America, and Johannesburg, South Africa | TF-CBT combined with art and play therapy, a pilot intervention for children that used both verbal and non-verbal therapy methods and alternated between a pre-determined structure and client-led expression. Group sessions included using ritual to create a safe place, using visual art materials and play therapy toys, engaging in psychoeducation through reading and responding to relevant books, learning how to identify feelings in self and others, and ending with a mindfulness practice. Three groups were also facilitated for the mothers during the course of the children’s intervention. | Intervention was once per week over 12 weeks and lasted 1-2 hours. | Safety; peer support; empowerment, voice and choice |
